# N-acetylglucosamine-Mediated Expression of *nagA* and *nagB* in *Streptococcus pneumoniae*

**DOI:** 10.3389/fcimb.2016.00158

**Published:** 2016-11-16

**Authors:** Muhammad Afzal, Sulman Shafeeq, Irfan Manzoor, Birgitta Henriques-Normark, Oscar P. Kuipers

**Affiliations:** ^1^Department of Molecular Genetics, Groningen Biomolecular Sciences and Biotechnology Institute, University of GroningenGroningen, Netherlands; ^2^Department of Bioinformatics and Biotechnology, Government College UniversityFaisalabad, Pakistan; ^3^Department of Microbiology, Tumor and Cell Biology, Karolinska InstitutetStockholm, Sweden

**Keywords:** N-acetylglucosamine, NAG, NagA, NagB, GlmS, pneumococcus, CcpA

## Abstract

In this study, we have explored the transcriptomic response of *Streptococcus pneumoniae* D39 to N-acetylglucosamine (NAG). Transcriptome comparison of *S. pneumoniae* D39 wild-type grown in chemically defined medium (CDM) in the presence of 0.5% NAG to that grown in the presence of 0.5% glucose revealed elevated expression of many genes/operons, including *nagA, nagB, manLMN*, and *nanP*. We have further confirmed the NAG-dependent expression of *nagA, nagB, manLMN*, and *nanP* by β-galactosidase assays. *nagA, nagB* and *glmS* are putatively regulated by a transcriptional regulator NagR. We predicted the operator site of NagR (*dre* site) in P*nagA*, P*nagB*, and P*glmS*, which was further confirmed by mutating the predicted *dre* site in the respective promoters (*nagA, nagB*, and *glmS*). Growth comparison of Δ*nagA*, Δ*nagB*, and Δ*glmS* with the D39 wild-type demonstrates that *nagA* and *nagB* are essential for *S. pneumoniae* D39 to grow in the presence of NAG as a sole carbon source. Furthermore, deletion of *ccpA* shows that CcpA has no effect on the expression of *nagA, nagB*, and *glmS* in the presence of NAG in *S*. *pneumoniae*.

## Introduction

Pneumonia, sepsis, meningitis, otitis media, and conjunctivitis are a few of the diseases caused by the major human pathogen *Streptococcus pneumoniae* that results in over a million deaths each year worldwide (Ispahani et al., 2004; O'Brien et al., 2009). *S. pneumoniae* relies on several nutrient sources and virulence factors to colonize in the human nasopharynx (Phillips et al., 1990; Titgemeyer and Hillen, 2002; Carvalho et al., 2011). Regulatory mechanisms of a number of carbon and nitrogen sources important for the lifestyle and virulence of *S. pneumoniae* have already been studied (Kloosterman et al., 2006b; Carvalho et al., 2011; Kloosterman and Kuipers, 2011; Afzal et al., 2015c,d). *S. pneumoniae* has been shown to metabolize 32 carbohydrates including the three-carbon molecule glycerol, nine hexoses or hexose derivatives (ascorbate, fructose, galactose, glucosamine, glucose, mannose, N-acetylglucosamine, N-acetyl-mannosamine, and N-acetyl-neuraminic acid), three α-galactosides (melibiose, raffinose, and stachyose), two β-galactosides (lactose, and lactulose), four α-glucosides (maltose, maltotriose, sucrose, and trehalose), seven β-glucosides (amygdalin, arbutin, 1-O-methyl-β-glucoside, cellobiose, gentiobiose, aesculin, and salicin) and six polysaccharides (glycogen, hyaluronate, inulin, maltodextrin, pectin, and pullulan) (Bidossi et al., 2012).

The importance of carbon sources in the life-style of *S. pneumoniae* can be judged from the fact that over 30% of all the transporters in the genome are presumably involved in sugar uptake (Tettelin et al., 2001; Bidossi et al., 2012), a considerably larger number than that present in the other microorganisms inhabiting the same niche (Paulsen et al., 2000; Tettelin et al., 2001). The glycoproteins lining the epithelial surfaces in the human nasopharynx might be good carbon and energy sources for pneumococcal growth. Notably, *S. pneumoniae* has the ability to grow on mucin as a sole carbon source (Yesilkaya et al., 2008). Mucins are constituents of the mucus that span the epithelial surfaces (Rose and Voynow, 2006). These structures are largely O-glycosylated glycoproteins and are usually composed of N-acetylglucosamine (NAG), N-acetylgalactosamine, N-acetylneuraminic acid, galactose, fucose, and sulphated sugars connected to the protein core, mostly *via* an N-acetylgalactosamine moiety (Rose and Voynow, 2006; Terra et al., 2010). *S. pneumoniae* has several extracellular glycosidases with an extensive variety of substrates specificities and can make use of the other host glycans, such as N-glycans and glycosaminoglycans (Burnaugh et al., 2008; King, 2010; Marion et al., 2012). These enzymes produce a number of free sugars that potentially can be used by the pneumococcus. The deglycosylation activity of both exo- and endoglycosidades has previously been demonstrated in *S. pneumoniae* (King et al., 2006; Burnaugh et al., 2008; Jeong et al., 2009; Marion et al., 2009). The ability to utilize complex glycans present at the site of colonization contributes to the successful survival and virulence of *S. pneumoniae* in the host (Buckwalter and King, 2012; Linke et al., 2013). Besides, the role of these enzymes in *in vivo* fitness is demonstrated by the findings that glycosidase mutants show attenuated capacity to colonize and to cause disease in mouse models (Tong et al., 2000; Jeong et al., 2009; Marion et al., 2009; Terra et al., 2010).

NAG is an important amino-carbon source for several bacteria due to its role as an energy resource and in peptidoglycan synthesis (Dobrogosz, 1968; Mobley et al., 1982). Several studies highlighted the importance of NAG as a preferred carbon source in bacteria (Dobrogosz, 1968; Mobley et al., 1982). The involvement of NAG for both catabolic and anabolic purposes requires proper regulatory mechanisms for its utilization, as shown in model microorganisms, such as *Bacillus subtilis, Escherichia coli, Streptococcus mutans*, and *Streptomyces coelicolor* (Plumbridge, 2001; Nothaft et al., 2010; Bertram et al., 2011; Zeng and Burne, 2015). The NAG regulon consists of *nagA, nagB*, and *glmS* in *S. mutans*, and is regulated by a GntR-family transcriptional regulator NagR (Moye et al., 2014). NagA is an NAG-6-phosphate deacetylase, whereas NagB is a GlcN-6-P deaminase, and GlmS is a Fru-6-P amidotransferase. NagB was upregulated in the presence of NAG while GlmS expression decreased, signifying that the regulatory mode of these enzymes depends on the concentration of environmental NAG. A *glmS*-inactivated mutant could not grow in the absence of NAG, whereas the growth of *nagB*-inactivated mutant was decreased in the presence of NAG (Kawada-Matsuo et al., 2012). *nagB* inactivation led to a decrease in the expression of virulence factors, including cell-surface protein antigen and glucosyltransferase, and also impeded biofilm formation and saliva-induced aggregation in *S. mutans* (Kawada-Matsuo et al., 2012). NagA has been shown to be important for the growth of pneumococcus in the presence of NAG as a sole carbon source (Paixão et al., 2015).

Diverse bacterial groups including streptomycetes, firmicutes, and enterobacteriaceae universally use phosphotransferase systems (PTSs) for uptake and phosphorylation of NAG (Simoni et al., 1976; Mobley et al., 1982; Alice et al., 2003; Nothaft et al., 2003, 2010). ManLMN PTS has been shown to be a glucose and mannose PTS in *Streptococcus salivarius* (Vadeboncoeur, 1984) and was also responsible for uptake of fructose, and NAG (Lortie et al., 2000). ManLMN transports glucose and mannose, and also shows specificity for galactose, NAG, and glucosamine in *S. pneumoniae* (Bidossi et al., 2012). NagP (PTS EIIBC component) is the main transporter of NAG in *B. subtilis* and *S. mutans* (Reizer et al., 1999; Saier et al., 2002; Moye et al., 2014).

Here, we demonstrate the effect of NAG on the global gene expression of *S. pneumoniae* and NAG-dependent expression of *nagA, nagB, manLMN*, and *nanP*. We further hypothesize that a GntR-family transcriptional regulator, NagR, might be involved in the regulation of *nagA, nagB*, and *glmS* and predict a putative operator site for NagR (*dre* site). We also explored the global impact of *ccpA* deletion on the transcriptome of *S. pneumoniae* in the presence of NAG, showing that *ccpA* has no effect on the expression of *nagA, nagB*, and *glmS*. Furthermore, we show that *nagA* and *nagB* are essential for *S. pneumoniae* to grow on NAG validating the previous study, where essentiality of *nagA* in the growth of *S. pneumoniae* was demonstrated (Paixão et al., 2015).

## Materials and methods

### Bacterial strains, growth conditions, and DNA isolation and manipulation

Bacterial strains and plasmids used in this study are listed in Table 1. *S. pneumonia*e D39 was grown as described previously (Kloosterman et al., 2006a; Afzal et al., 2014). For β-galactosidase assays, derivatives of *S. pneumoniae* D39 were grown in chemically defined medium (CDM) (Neves et al., 2002) supplemented either with 0.5% glucose or with 0.5% NAG. For selection on antibiotics, medium was supplemented with the following concentrations of antibiotics: 150 μg/ml spectinomycin, 15 μg/ml trimethoprim and 2.5 μg/ml tetracycline for *S. pneumoniae*; and 100 μg/ml ampicillin for *E. coli*. All bacterial strains used in this study were stored in 10% (v/v) glycerol at −80°C. For PCR amplification, chromosomal DNA of *S. pneumoniae* D39 (Lanie et al., 2007) was used. Primers used in this study are based on the sequence of the *S. pneumoniae* D39 genome and listed in Table 2.

**Table 1:** **List of strains and plasmids used in this study**.

**Strain/plasmid**	Description^*^	**Source**
***S. PNEUMONIAE***		
D39	Serotype 2 strain	Laboratory of P. Hermans
MA700	D39 Δ*nagA*; Trm^R^	This study
MA701	D39 Δ*nagB*; Spec^R^	This study
MA702	D39 Δ*glmS*	This study
MA703	D39 Δ*bgaA*::P*nagA*-*lacZ*; Tet^R^	This study
MA704	D39 Δ*bgaA*::P*nagB*-*lacZ*; Tet^R^	This study
MA705	D39 Δ*bgaA*::P*glmS*-*lacZ*; Tet^R^	This study
MA706	D39 Δ*bgaA*::P*manL*-*lacZ*; Tet^R^	This study
MA707	D39 Δ*bgaA*::P*nagA*-*M*-*lacZ*; Tet^R^	This study
MA708	D39 Δ*bgaA*::P*nagB*-*M*-*lacZ*; Tet^R^	This study
MA709	D39 Δ*bgaA*::P*glmS1*-*M*-*lacZ*; Tet^R^	This study
MA710	D39 Δ*bgaA*::P*glmS3*-*M*-*lacZ*; Tet^R^	This study
MA203	D39 Δ*bgaA*::P*nanE*-*lacZ*; Tet^R^	Afzal et al., 2015b
***E. COLI***		
EC1000		Laboratory collection
**PLASMIDS**		
pPP2	Amp^R^ Tet^R^; promoter-less *lacZ*. For replacement of *bgaA* with promoter *lacZ* fusion. Derivative of pPP1	Halfmann et al., 2007
pORI280	Erm^R^; *ori*^+^ *repA*^−^*;* deletion derivative of pWV01; constitutive *lacZ* expression from P32 promoter	Leenhouts et al., 1998
pMA700	pORI280 carrying *glmS* deletion	This study
pMA701	pPP2 P*nagA-lacZ*	This study
pMA702	pPP2 P*nagB-lacZ*	This study
pMA703	pPP2 P*glmS-lacZ*	This study
pMA704	pPP2 P*manL-lacZ*	This study
pMA705	pPP2 P*nagA-M-lacZ*	This study
pMA706	pPP2 P*nagB-M-lacZ*	This study
pMA707	pPP2 P*glmS1-M-lacZ*	This study
pMA708	pPP2 P*glmS3-M-lacZ*	This study

* *Amp^R^*, Spec^R^, Tet^R^, and Trm^R^ confer ampicillin, spectinomycin, tetracycline and trimethoprim resistance gene, respectively.

**Table 2:** **List of primers used in this study**.

**Name**	**Nucleotide sequence (5′ 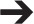 3′)^*^**	**Restriction site**
nagA-R	CATGGGATCCGTCCACAAGTTCCAAGTAACC	*BamHI*
nagA-F	CATGGAATTCGCAGACAGCTCAAGACAAGC	*EcoRI*
nagA-F-M	CATGGAATTCTATCTCCAAAAAATAGGTCGCTGTCATTTACAAAT	*EcoRI*
nagB-R	CATGGGATCCGCAACTTTTCCACCTTGAACTTGG	*BamHI*
nagB-F	CATGGAATTCGGGCAATCAATTCCTCTGGC	*EcoRI*
nagB-F-M	CATGGAATTCCGTTTTCACTTGACAAAAATTGGTCGCTGTCATATAATAA	*EcoRI*
glmS-R	CATGGGATCCCAACACCAACAATTCCACAC	*BamHI*
glmS-F	CATGGAATTCCGTCGTCTGAAGAAATCAGG	*EcoRI*
manL-F	CATGGAATTCCAGTAGAAGATGCTGTTG	*EcoRI*
manL-R	CATGGGATCCTGACTGATGAATACCC	*BamHI*
glmS1-F-M	CATGGAATTCACAGGAGCTTAATTTGAACGCTGTCAATTTTTACTC	*EcoRI*
glmS3-R-M	CATGGGATCCCACATAGTATATACGACACAGGCAAGCTGTGCTTTCTCCTTAAAATTGGGCGCGTCTAATTCA	*BamHI*
nagA-1	GACGGTGGTCATTGCGACTG	–
nagA-2	GCATAGGCGCGCCCCTCGACGAACTCCGTGTG	*AscI*
nagA-3	CGATTGCGGCCGCGGTAGCAACCTACCTAGATGG	*NotI*
nagA-4	CGTAGATATTCAGCCTGCATACC	–
nagB-1	GGGTGTCGTTCATGACAAGGG	–
nagB-2	GCATAGGCGCGCCGCTACTTCCTGTCGCAAGTCC	*AscI*
nagB-3	CGATTGCGGCCGCGCAGATGCTGAAGCGCTTAGC	*NotI*
nagB-4	CCATAGACAATGTCTAGTCTAAGC	–
glmS-1	TGCTCTAGAGGTCATCTTCGTGAACTTCACCG	*XbaI*
glmS-2	CCGCAGAATCATAGCCACGG	–
glmS-3	GCTATGATTCTGCGGCGACTGTACACCCTTACCTCTC	–
glmS-4	GAAGATCTCCAGGACAATCTCTGGGGC	*BglII*
Spec-R	GCTAAGCGGCCGCACTAAACGAAATAAACGC	*NotI*
Spec-F	GCTATGGCGCGCCCTAATCAAAATAGTGAGGAGG	*AscI*
Trmp-R	GCATGCGGCCGCGTTACGACGCGCATAGACGG	*AscI*
Trmp-F	GCATGGCGCGCCGGATTTTTGTGAGCTTGGA	*NotI*

* The underlined sequences represent the respective restriction sites.

### Construction of *nagA, nagB*, and *glmS* mutants

*nagA* and *nagB* deletion mutants were made by allelic replacement with trimethoprim- and spectinomycin-resistance cassettes, respectively. Briefly, primers nagA-1/nagA-2 and nagA-3/nagA-4 were used to generate PCR fragments of the left and right flanking regions of *nagA.* Similarly, primers nagB-1/nagB-2 and nagB3/nagB-4 were used to generate PCR fragments of the left and right flanking regions of *nagB*. PCR products of left and right flanking regions of *nagA* and *nagB* contain *AscI* and *NotI* restriction enzyme sites, respectively. The trimethoprim- and spectinomycin-resistance cassettes that are amplified by primers Spec-F/Spec-R and Trmp-F/Trmp-R, respectively from pORI38 and pKOT, also contain *AscI* and *NotI* restriction enzyme sites on their ends. Then, by restriction and ligation, the left and right flanking regions of *nagA* and *nagB* were fused to the trimethoprim- and spectinomycin-resistance genes, respectively. The resulting ligation products were transformed to *S. pneumoniae* D39 wild-type and selection of the mutant strains was done on appropriate concentrations of trimethoprim and spectinomycin.

A markerless *glmS* mutant (Δ*glmS*) was constructed in the *S. pneumoniae* D39 wild-type using the pORI280 plasmid, as described before (Kloosterman et al., 2006a). Primer pairs, glmS-1/glmS-2 and glmS-3/glmS-4, were used to generate PCR fragments of the left and right flanking regions of *glmS*, respectively. These PCR fragments were inserted into pORI280 using *XbaI* and *BglII* restriction sites, resulting in pMA700. All mutants were further confirmed by PCR and DNA sequencing.

### Growth analysis

For growth analysis of Δ*nagA*, Δ*nagB*, and Δ*glmS, S. pneumoniae* D39 wild-type and its isogenic mutants (Δ*nagA*, Δ*nagB*, and Δ*glmS*) were grown microaerobically at 37°C in 5 ml tubes containing 3 ml CDM supplemented either with 0.5% NAG or with 0.5% Glucose. Cultures were maintained at 37°C for 11 h and optical density at 600 nm was recorded with 1 h interval. CDM without inoculum was taken as blank. The growth of each strain was monitored from six biological replicates from at least two different days.

### Construction of promoter *lacZ*-fusions and β-galactosidase assays

Chromosomal transcriptional *lacZ*-fusions to the *nagA, nagB, glmS*, and *manL* promoters were constructed in the integration plasmid pPP2 (Halfmann et al., 2007) with primer pairs mentioned in Table 2, resulting in pMA701-04 respectively. Briefly, PCR products of *nagA, nagB, glmS*, and *manL* promoters were obtained using primers pairs mentioned in Table 2. These PCR fragments contain *EcoRI* and *BamHI* restriction sites at their ends. pPP2 also has *EcoRI* and *BamHI* restriction sites in its multiple cloning site (MCS). Then, by restriction and ligation, these PCR fragments were cloned into pPP2. pMA701-04 were further introduced into the *S. pneumoniae* D39 wild-type resulting in strains MA703-06, respectively. All plasmid constructs were checked by PCR and DNA sequencing. β-galactosidase assays were performed as described before (Israelsen et al., 1995; Kloosterman et al., 2006a) using cells that were grown in CDM with appropriate sugar mentioned in Results section and harvested in the mid-exponential phase of growth.

To study the functionality of *dre* site, the following promoter *lacZ*-fusions of *nagA, nagB*, and *glmS* with mutated *dre* sites were made in pPP2 (Halfmann et al., 2007) using the primer pairs mentioned in Table 2: P*nagA-M* (mutation in the *dre* site), P*nagB-M* (mutation in the *dre* site), P*glmS1-M* (mutation in the 1st *dre* site), and P*glmS3-M* (mutation in the 3rd *dre* site), resulting in plasmid pMA705-08, respectively. The mutations were incorporated into the primers used to amplify the target promoter regions containing the *dre* sites. These constructs were introduced into the *S. pneumoniae* D39 wild-type, resulting in strains MA707-10.

### Microarray analysis

For DNA microarray analysis in the presence of NAG, the transcriptome of *S. pneumoniae* D39 wild-type, grown in biological replicates in CDM with 0.5% NAG was compared to D39 wild-type grown in CDM with 0.5% glucose. Similarly, for DNA microarray analysis of Δ*ccpA*, the transcriptome of *S. pneumoniae* D39 wild-type was compared to D39 Δ*ccpA*, grown in biological replicates in CDM with 0.5% NAG. The cells were harvested at their respective mid-exponential growth phases. All other procedures regarding the DNA microarray experiment and data analysis were performed as previously described (Afzal et al., 2015a; Shafeeq et al., 2015). For the identification of differentially expressed genes a Bayesian *p* < 0.001 and a fold change cut-off ≥2 was applied. Microarray data have been submitted to GEO (Gene Expression Omnibus) database under the accession number GSE89589 and GSE89590.

## Results

### The putative NAG regulon in *S. pneumoniae*

The NAG regulon consists of *nagA, nagB*, and *glmS* in *S. mutans* and is regulated by a GntR-family transcriptional regulator NagR (Moye et al., 2014). NagA is an NAG-6-phosphate deacetylase, whereas NagB is a GlcN-6-P deaminase, and GlmS is a Fru-6-P amidotransferase. *S. pneumoniae* D39 also possess the genes that encode proteins putatively involved in the transport and utilization of NAG. These genes are *nagA, nagB, manLMN, nanP*, and *glmS*. In *S. pneumoniae*, it appears that NAG enters the cell through NanP PTS (SPD-1496) and/or ManLMN (SPD-0262-64) and is subsequently phosphorylated (Kanehisa et al., 2014). NanP (PTS) is encoded by the gene that is part of *nan* operon-I (*spd_1488-97*) of the *nan* gene cluster and is proposed to transport amino sugars (Bidossi et al., 2012; Afzal et al., 2015b). *nanP* codes for EIIBC components of the PTS and therefore, needs EIIA component of another PTS to phosphorylate the incoming NAG. The phosphorylated NAG is deacetylated to glucosamine-6-P by NagA (Kanehisa et al., 2014). NagB converts glucosamine-6-P to fructose-6-P, whereas GlmS converts fructose-6-P to glucosamine-6-P. The role of NAG on the gene expression of *S. pneumoniae* has not been investigated before. Therefore, we decided to explore the effect of NAG on the whole transcriptome of *S. pneumoniae*.

### NAG-dependent gene expression in *S. pneumoniae*

To study the transcriptomic response of *S. pneumoniae* D39 to NAG, microarray comparison of *S. pneumoniae* D39 wild-type grown in CDM with 0.5% NAG to that grown in CDM with 0.5% glucose was performed. Presence of NAG in the medium resulted in altered expression of a number of genes/operons after applying the criteria of ≥2.0-fold and *p* < 0.001 (Table S1). Table 3 summarizes the transcriptome changes incurred in *S. pneumoniae* in the presence of NAG and lists the fold-change in the expression of the putative NAG transport and utilization genes.

**Table 3:** **Summary of data from Table S1 showing transcriptome comparison of *S. pneumoniae* D39 wild-type grown in CDM with 0.5% NAG to that grown in CDM with 0.5% glucose**.

**D39 tag^a^**	**Function^b^**	**Ratio^c^**
*spd_1971*	Glycosyl hydrolase-related protein	22.8
*spd_0063*	β-N-acetylhexosaminidase, StrH	14.2
*spd_1970*	ROK family protein	13.9
*spd_1050*	Tagatose 1,6-diphosphate aldolase, LacD	13.7
*spd_1972*	hypothetical protein	13.6
*spd_0277*	6-phospho-β-glucosidase	13.1
*spd_1969*	Glycosyl hydrolase-related protein	11.9
*spd_1051*	Tagatose-6-phosphate kinase, LacC	11.0
*spd_1052*	Galactose-6-phosphate isomerase, LacB	10.7
*spd_1053*	Galactose-6-phosphate isomerase, LacA	9.9
*spd_0280*	Transcriptional regulator, CelR	7.7
*spd_1677*	Sugar ABC transporter, RafE	7.4
*spd_1676*	Sugar ABC transporter, RafF	6.6
*spd_1634*	Galactokinase, GalK	6.5
*spd_1633*	Galactose-1-phosphate uridylyltransferase, GalT	6.3
*spd_1675*	Sugar ABC transporter, RafG	5.6
*spd_1047*	PTS system, lactose-specific IIBC components, LacE	5.4
*spd_0283*	PTS system, IIC component	5.2
*spd_1974*	Hypothetical protein	4.8
*spd_1046*	6-phospho-β-galactosidase, LacG	4.4
*spd_1049*	Transcription antiterminator, LacT	4.4
*spd_0282*	Hypothetical protein	4.4
*spd_0281*	PTS system, IIA component	4.2
*spd_1664*	PTS system, trehalose-specific IIABC components	4.2
*spd_1663*	α-phosphotrehalase, TreC	4.0
*spd_0279*	PTS system, IIB component	3.9
*spd_1495*	Sugar ABC transporter, sugar-binding protein	3.7
*spd_1973*	α-1,2-mannosidase, putative	3.6
*spd_1496*	PTS system, IIBC components	3.4
*spd_0263*	PTS system, mannose-specific IIC component, ManM	3.2
*spd_0262*	PTS system, mannose/fructose/sorbose family protein, IID component	3.0
*spd_1494*	Sugar ABC transporter, permease protein	2.7
*spd_1493*	Sugar ABC transporter, permease protein	2.4
*spd_1866*	N-acetylglucosamine-6-phosphate deacetylase, NagA	2.4
*spd_1846*	PTS system, IIB component	2.3
*spd_1246*	glucosamine-6-phosphate isomerase, NagB	2.3
*spd_0264*	PTS system, mannose-specific IIAB components, ManL	2.1
*spd_1492*	Hypothetical protein	2.0
*spd_1491*	Hypothetical protein	2.0
*spd_1100*	Glucose-6-phosphate 1-dehydrogenase, Zwf	−2.2
*spd_0448*	Glutamine synthetase, GlnA	−3.3
*spd_1099*	Amino acid ABC transporter, ATP-binding protein	−3.3
*spd_0447*	Transcriptional regulator, GlnR	−3.7
*spd_1098*	Amino acid ABC transporter, amino acid-binding protein	−4.4

a Gene numbers refer to D39 locus tags.

b D39 annotation (Lanie et al., 2007).

c Ratio represents the fold increase/decrease in the expression of genes in the presence of 0.5% NAG compared to 0.5% glucose.

The glutamine regulon was downregulated around 4-fold in the presence of NAG. This regulon consists of genes involved in glutamine synthesis and uptake (*glnA* and *glnPQ*), glutamate synthesis (*gdhA*), and the gene coding for the pentose phosphate pathway enzyme Zwf, which forms an operon with *glnPQ* (Kloosterman et al., 2006b). The glutamine regulon is shown to be repressed in the presence of a nitrogen source (Kloosterman et al., 2006b). The presence of nitrogen in NAG might be the reason of down-regulation of the glutamine regulon. A putative operon *spd_1969-72* was highly upregulated in the presence of NAG. This operon encodes proteins that are putatively involved in the utilization of carbohydrates. *spd_1970* codes for a ROK-family protein (RokD), but it lacks an HTH (helix-turn-helix) domain making it a less probable candidate as a transcriptional regulator of this operon (Shafeeq et al., 2012). ROK-family proteins are a class of transcriptional regulators involved in carbohydrate-dependent transcriptional control (repressor, ORF and kinase). They also contain sugar kinases and many functionally uncharacterized proteins (Titgemeyer et al., 1994). This operon was also upregulated in the presence of cellobiose (Shafeeq et al., 2013) and some other sugars making it a candidate for the utilization of multiple sugars. *strH* is another gene that was highly upregulated in the presence of NAG. StrH is a β-N-acetylhexosaminidase and is an important virulence factor of *S. pneumoniae*. StrH is a cell-surface attached β-N-acetylglucosaminidase that is used by *S. pneumoniae* to process the termini of host complex N-linked glycans (Pluvinage et al., 2013). StrH and SPD-1969 are also possibly being involved in the conversion of chitobiose into NAG based presumably on bioinformatics (Kanehisa et al., 2014). Similarly, *spd_1973-74* was also upregulated in our microarray analysis. Both these genes are annotated to be involved in carbohydrate metabolism, where SPD-1973 is a putative α-1,2-mannosidase and SPD-1974 is a hypothetical protein.

*cel* gene cluster (*spd*-*0277*-*0283*) putatively involved in the utilization of cellobiose is upregulated in the presence of NAG. This gene cluster is shown to be activated by transcriptional regulator CelR in the presence of cellobiose (Shafeeq et al., 2011). Tagatose pathways (*lac* gene cluster) and Leloir pathway genes (*galK* and *galT*) involved in the utilization of lactose and galactose (Afzal et al., 2014, 2015e) are significantly upregulated in the presence of NAG. *lac* gene cluster comprises of two operons in *S. pneumoniae*, i.e., *lac* operon-I (*lacABCD*) and *lac* operon-II (*lacFEG*) (Afzal et al., 2014). LacR, a DeoR-type regulator acts as a transcriptional repressor of *lac* operon-I in the absence of lactose/galactose (Afzal et al., 2014). Whereas, BglG-family transcriptional antiterminator LacT acts as a transcriptional activator of the *lac* operon-II in the presence of lactose (Afzal et al., 2014). Putative Raffinose uptake genes *rafEFG* (*spd*-*1675*-*77*) are highly upregulated in the presence of NAG. Glucose and sucrose are shown to inhibit raffinose uptake (Tyx et al., 2011). A putative trehalose system (*spd*-*1663*-*64*) is highly expressed under our tested conditions. Upregulation of these different sugar systems under our tested conditions might be due to absence of CCR in the presence of NAG as a sole carbon source and further experiments are required to explore the role of these genes in the utilization of NAG.

Expression of genes that are putatively part of NAG utilization and transport pathway was also altered in our transcriptome analysis (Table 3). Expression of *manLMN* is upregulated around 3-fold in the presence of NAG. We observed upregulation of the *nan* operon-I, which is involved in the transport and utilization of sialic acid (an amino carbon source) (Marion et al., 2011). Moreover, expression of *nagA* and *nagB* was upregulated more than two folds in the presence of NAG (Table 3). No change in the expression of *glmS* is observed in our transcriptome in the presence of NAG. Upregulation of *nanP, manLMN, nagA*, and *nagB* in our transcriptome supports that these genes are important in NAG utilization in *S. pneumoniae* and strengthens the notion that NanP and ManLMN might be very important for NAG transport. Therefore, we decided to further explore the regulation of these genes in the presence of NAG.

### NAG induces the expression of the genes involved in the putative transport and utilization of amino sugars

**Figure 1:**
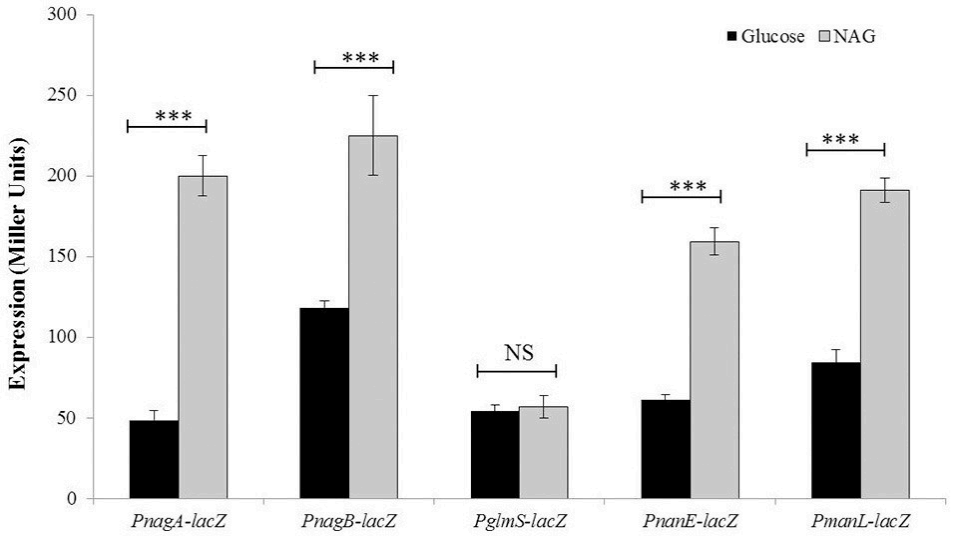
**Expression levels (in Miller units) of P*nagA-lacZ*, P*nagB-lacZ*, P*glmS*-*lacZ*, P*nanE*-*lacZ*, and P*manL*-*lacZ* in *S. pneumoniae* D39 wild-type grown in CDM either with 0.5% glucose or with 0.5% NAG**. Standard deviations of three independent experiments are indicated in bars. Statistical significance of the differences in the expression levels was determined by one-way ANOVA (NS, not significant and ^***^P < 0.0001).

In order to investigate in more detail the transcriptional regulation of *nanP, manLMN, nagA*, and *nagB* in the presence of NAG and to confirm our microarray results, we made ectopic transcriptional *lacZ*-fusions of *nanE, manL, nagA*, and *nagB* promoters (P*nagA-lacZ*, P*nagB-lacZ*, P*nanE*-*lacZ*, and P*manL*-*lacZ*) and performed β-galactosidase assays (Figure 1). Our β-galactosidase assays data revealed that the expression of P*nagA-lacZ*, P*nagB-lacZ*, P*nanE*-*lacZ*, and P*manL*-*lacZ* was strikingly higher in the presence of NAG compared to glucose in CDM (Figure 1). These data further confirm our microarray data mentioned above. We did not observe any change in the expression of *glmS* in our microarray analysis in the presence of NAG. To further study the expression of *glmS* in the presence of NAG and confirm our microarray analysis, we constructed ectopic transcriptional *lacZ*-fusion of *glmS* promoter (P*glmS*-*lacZ*) and performed β-galactosidase assays. We could not see any significant change in the expression of P*glmS-lacZ* under our tested conditions.

### Predication and confirmation of the *dre* sites in the promoter regions of *glmS, nagA*, and *nagB*

Recently, NagR was characterized as a transcriptional repressor and shown to bind with specific DNA sequences named as *dre* sites, present in the promoter regions of the *nagAB* and *glmS* genes in *S. mutans* (Zeng and Burne, 2015). Blast search in *S. pneumoniae* D39 for NagR revealed the presence of a putative GntR-family transcriptional regulator NagR (SPD-1275) in *S. pneumoniae* D39. Presence of a NagR ortholog in *S. pneumoniae* might suggest its role in the regulation of the *glmS, nagA*, and *nagB*. We decided to delete *nagR* in *S. pneumoniae* D39 and study its role. However, we could not delete *nagR* in *S. pneumoniae* D39, suggesting the essentiality of NagR in the lifestyle of *S. pneumoniae*.

To explore the NagR regulated genes in *S. pneumoniae* D39, we decided to explore the genome of *S. pneumoniae* D39 for *dre* sites by using the Genome2D tool (Baerends et al., 2004) and a MEME motif sampler search (Bailey and Elkan, 1994). A 20-bp consensus sequence was found upstream of *nagA* (5′-AAATAGGTCTATACCATTTA-3′) and *nagB* (5′- AAATTGGTCTATACCATATA-3′) in *S. pneumoniae* D39 (Figure S1). We also found three *dre* sites in the promoter region of *glmS* (5′-AATTTGAACTATACCAATTT-3′, 5′-AAACAAGTATATACTGTTTT-3′ and 5′-GAATTAGACTATACCAATTT-3′). These DNA stretches may serve as *dre* sites in *S. pneumoniae*. We further checked the conservation of this *dre* site in other streptococcal species (*Streptococcus mitis, Streptococcus agalactiae, Streptococcus dysgalactiae, Streptococcus equi, S. mutans, Streptococcus pyogenes, Streptococcus sanguinis, Streptococcus gallolyticus, Streptococcus suis*, and *Streptococcus uberis*) and constructed weight matrix of the putative *dre* sites presents in different streptococci (Figure S2). We found that the *dre* sequence is highly conserved in the promoter regions of *nagA, nagB* and *glmS* in these streptococci as well (Figure S1).

**Figure 2:**
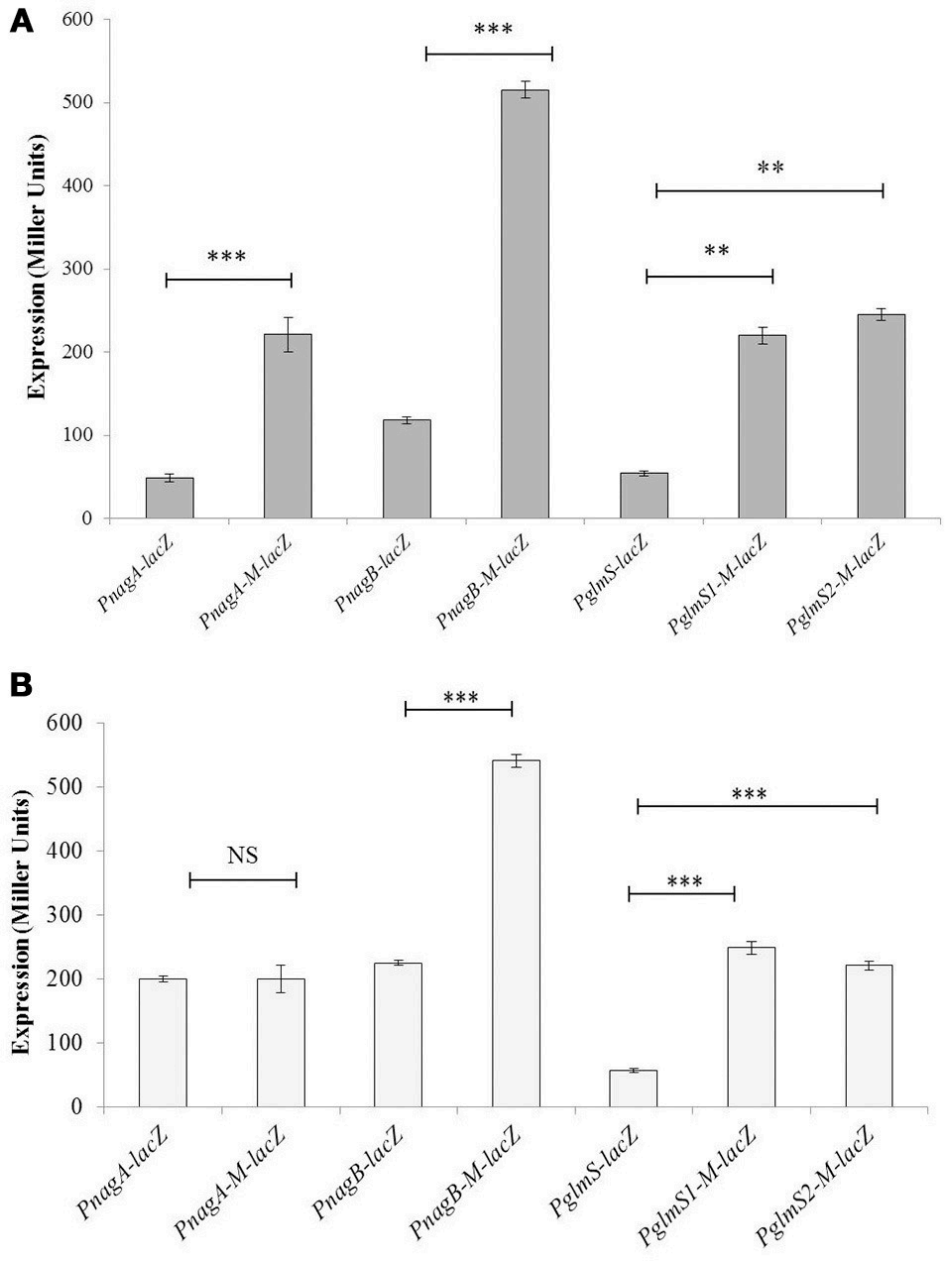
**Verification of *dre* sites in P*nagA*, P*nagB*, and P*glmS.*** Expression levels (in Miller units) of P*nagA-lacZ*, P*nagB-lacZ*, P*glmS*-*lacZ*, P*nagA-M-lacZ*, P*nagB-M-lacZ*, P*glmS1*-*M*-*lacZ*, and P*glmS3-M*-*lacZ* in *S. pneumoniae* D39 wild-type grown in CDM with 0.5% glucose **(A)** and 0.5% NAG **(B)**. P*nagA-M-lacZ* and P*nagB-M-lacZ* represent promoter *lacZ*-fusions of *nagA* and *nagB* with mutated *dre* sites, whereas P*glmS1-M*-*lacZ* and P*glmS3-M*-*lacZ* represents promoter-*lacZ*-fusions with mutated *dre* site 1 and 3, respectively in P*glmS*. Standard deviations of three independent experiments are indicated in bars. Statistical significance of the differences in the expression levels was determined by one-way ANOVA (NS, not significant, ^**^P < 0.001, and ^***^P < 0.0001).

To determine if the located stretch of DNA mediates the NagR-dependent transcriptional control of the *glmS, nagA*, and *nagB*, we made a number of transcriptional *lacZ*-fusions, where conserved bases in the putative *dre* sites were mutated in P*nagA* (5′-AAATAGGTCTATACCATTTA-3′ to 5′-AAATAGGTC**GC**T**GT**CATTTA-3′), P*nagB* (5′-AAATTGGTCTATACCATATA-3′ to 5′-AAATTGGTC**GC**T**GT**CATATA-3′) and P*glmS* (first site: 5′-AATTTGAACTATACCAATTT-3′ to 5′-AATTTGAAC**GC**T**GT**CAATTT-3′ and third site: 5′-GAATTAGACTATACCAATTT-3′ to 5′-GAATTAGAC**GCGC**CCAATTT-3′). We could not mutate the second *dre* site in P*glmS* as it overlaps with core promoter sequence. The expression of P*nagA*-*M*-*lacZ* and P*nagB*-*M*-*lacZ* (few conserved bases of the *dre* sites were mutated) compared to that of the promoters with the intact *dre* sites (P*nagA*-*lacZ* and P*nagB*-*lacZ*) was considerably higher in the presence of glucose and NAG (Figures 2A,B). A derepression of the expression of P*glmS* was observed when either of the putative *dre* sites in P*glmS* (*dre* site 1 and 3) was mutated. These results suggest that *dre* sites present in P*glmS*, P*nagA* and P*nagB* are active and may act as an operator site for NagR in *S. pneumoniae*.

### *nagA* and *nagB* are essential for *S. pneumoniae* D39 to grow in the presence of NAG as a sole carbon source

**Figure 3:**
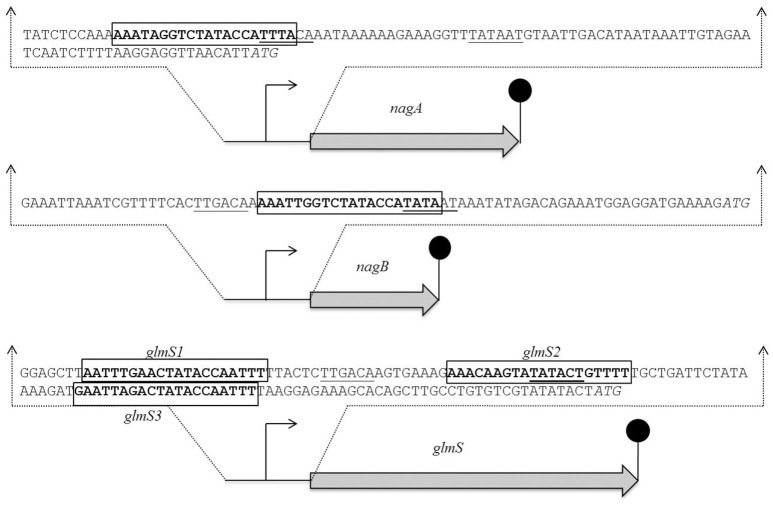
**Identification of the *dre* site in the promoter regions of *nagA*, *nagB*, and *glmS* in *S. pneumoniae* D39 wild-type**. Translational start sites are italicized and putative *dre* sites are bold and rectangle. Core promoter sequences are underlined.

**Figure 4:**
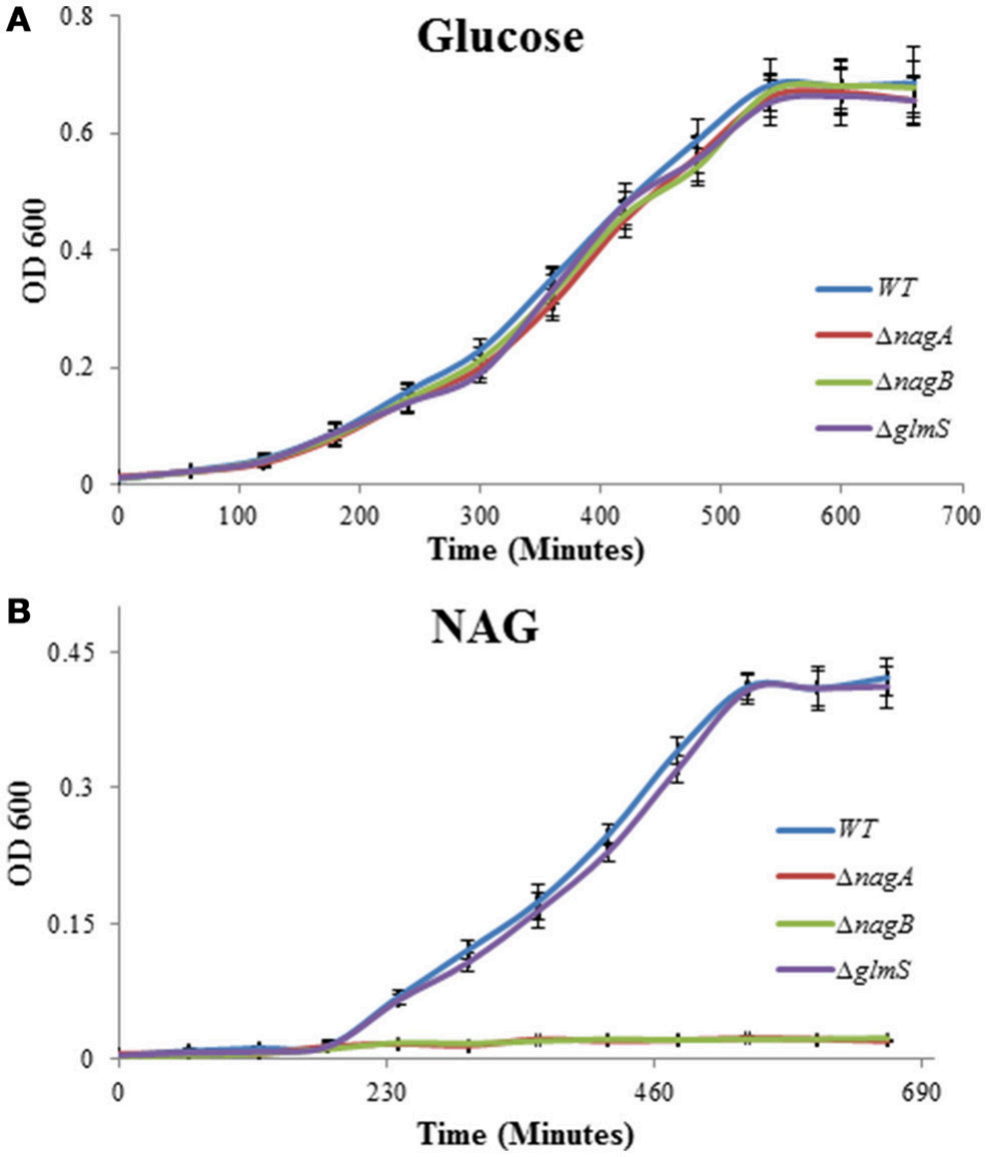
**Growth of *S. pneumoniae* D39 wild-type and its isogenic mutants Δ*nagA*, Δ*nagB*, and Δ*glmS* grown in CDM with 0.5% glucose (A)** and 0.5% NAG **(B)**.

*nagA, nagB*, and *glmS* encode important enzymes for the metabolism of NAG in bacteria. To elucidate the significance of these genes on the growth of *S. pneumoniae*, we made knockout mutants of these genes (Δ*nagA*, Δ*nagB*, and Δ*glmS*), and explored the impact of mutation of these genes on the growth of *S. pneumoniae* D39 in the presence of 0.5% NAG or glucose in CDM. The genetic organization and PCR confirmation of *nagA, nagB*, and *glmS* mutants are given in the Figure 3 and Figure S3, respectively. All three mutants (Δ*nagA*, Δ*nagB*, and Δ*glmS*) had approximately the same growth as D39 wild-type in the presence of glucose in the medium (Figure 4). Δ*glmS* also showed the same growth pattern as the D39 wild-type in the presence of NAG. However, in contrast to D39 wild-type, Δ*nagA* and Δ*nagB* were not able to grow in the presence of NAG (Figure 4). These results suggest that *nagA* and *nagB* are necessary for *S. pneumoniae* to grow in the presence of NAG. These results are also in accordance with recently published data, where they showed that a mutant of *nagA* did not grow in the presence of NAG as a sole carbon source (a phenotype that could be complemented) (Paixão et al., 2015).

### Role of CcpA in regulation of *nagA, nagB, nanP, glmS*, and *manL*

CcpA is the master transcriptional regulator in *S. pneumoniae* that represses the expression of genes involved in the utilization of non-preferred sugars in the presence of a preferred one. To explore the effect of *ccpA* deletion on the transcriptome of *S. pneumoniae* and in NAG-dependent regulation of NAG utilization and transport genes, we performed transcriptome comparison of D39 Δ*ccpA* to D39 wild-type in CDM with 0.5% NAG. Expression of a number of genes was altered significantly (Table S1). These genes have been categorized according to their protein function in COG categories (Table 4). We did not observe any significant change in the expression of *nagA, nagB*, or *glmS*, suggesting CcpA independent expression of these genes. However, expression of *manLMN* and *nan* operon-I was upregulated in Δ*ccpA*, which might suggest a putative role of CcpA in regulation of the *manLMN* and *nan* operon-I. *nan* operon-I was already shown to be regulated by CcpA and to have a *cre* box (Afzal et al., 2015b). Therefore, upregulation of *nan* operon-I in the absence of *ccpA* strengthens the previous observation (Afzal et al., 2015b).

**Table 4:** **Number of genes significantly affected in *S. pneumoniae* D39 Δ*ccpA* compared to the D39 wild-type grown in CDM with 0.5% NAG**.

**Functional categories**	**Total**	**Up**	**Down**
C: Energy production and conversion	10	4	6
D: Cell cycle control, cell division, chromosome partitioning	2	0	2
E: Amino acid transport and metabolism	4	3	1
F: Nucleotide transport and metabolism	1	0	1
G: Carbohydrate transport and metabolism	19	17	2
H: Coenzyme transport and metabolism	1	0	1
I: Lipid transport and metabolism	3	0	3
J: Translation, ribosomal structure and biogenesis	15	3	12
K: Transcription	3	2	1
L: Replication, recombination and repair	3	2	1
M: Cell wall/membrane/envelope biogenesis	6	2	4
O: Posttranslational modification, protein turnover, chaperones	3	3	0
P: Inorganic ion transport and metabolism	3	1	2
Q: Secondary metabolites biosynthesis, transport and catabolism	2	1	1
R: General function prediction only	5	3	2
S: Function unknown	42	22	20
T: Signal transduction mechanisms	3	3	0
U: Intracellular trafficking, secretion, and vesicular transport	2	2	0
V: Defense mechanisms	8	7	1
Others	34	19	15
Total number of genes	169	94	75

Genes affected with more than 2-fold in D39 ΔccpA compared to the D39 wild-type are shown in COG functional categories.

To further confirm the role of CcpA in the regulation of *nagA, nagB, glmS*, and *manL*, we analyzed the promoter regions of these genes for the presence of *cre* boxes. We could not find a *cre* box in the promoter regions of *nagA, nagB*, and *glmS*, which might confirm the CcpA-independent regulation of the *nagA, nagB*, and *glmS* by transcriptional regulator NagR. However, we found a putative *cre* box (5′-ATGAAAACGGTTTATA-3′) in the promoter regions of *manL*, further confirming the role of CcpA in the regulation of *manLMN*.

## Discussion and conclusions

The existence of well-developed sugar transport mechanisms in the opportunistic respiratory human pathogen, *S. pneumoniae*, emphasizes the importance of carbohydrates in the lifestyle of pneumococcus and confers an extra advantage to survive in a changing nutritional environment (Tettelin et al., 2001). Glucose is the most preferred carbon source for *S. pneumoniae* but the presence of several other sugar-specific systems in *S. pneumoniae* indicates its ability to use other available sugars (Hoskins et al., 2001; Lanie et al., 2007; Bidossi et al., 2012). Extensive studies have been performed regarding regulatory mechanisms of different dedicated systems for sugars, including maltose, raffinose, cellobiose, sialic acid, and others in *S. pneumoniae* (Tyx et al., 2011; Shafeeq et al., 2013; Afzal et al., 2015b,f). Lack of free carbohydrates in the human airway makes modification and import of complex glycans much more critical for pneumococci to obtain the necessary carbon (Buckwalter and King, 2012). At least nine surface-associated glycosidases have been shown to modify host glycans in pneumococci, which makes bacterial survival better in the host (King et al., 2006; Burnaugh et al., 2008; Dalia et al., 2010). Data suggests that NAG may be an important carbohydrate for pneumococci (Bidossi et al., 2012). The regulatory mechanisms of genes putatively involved in NAG utilization have not been explored in *S. pneumoniae*. The current study sheds light on the regulatory mechanism of the *nagA, nagB*, and *glmS* in *S. pneumoniae*.

*nagA, nagB*, and *glmS* are annotated as a part of the amino sugar metabolism pathways in *S. pneumoniae* (Kanehisa et al., 2014). In our transcriptome comparison of *S. pneumoniae* D39 grown in CDM with 0.5% NAG to that grown in CDM with 0.5% glucose revealed increased expression of *nagA, nagB, manLMN*, and *nanP*. In *S. mutans*, expression of *glmS* is repressed in the presence of NAG compared to glucose (Zeng and Burne, 2015). This repression of *glmS* in the presence of NAG was relieved in *nagR* mutant (Zeng and Burne, 2015). However, no change in the expression of *glmS* is observed in our NAG-dependent transcriptome and no effect of *ccpA* deletion on the expression of *glmS* is observed. Mutating *dre* site 1 or 3 in the P*glmS* led to increase in expression of *PglmS* in the presence of glucose and NAG. This might indicate that NagR represses the expression of *glmS* in the presence of glucose and NAG.

The transport of amino-sugars has been attributed to a PTS (NanP) and *manLMN* in *S. mutans* (Moye et al., 2014). Similarly, a NAG-specific PTS (NagE) and a mannose-specific PTS ManXYZ have been shown to be involved in the NAG transport in *E. coli* (White, 1970; Alvarez-Añorve et al., 2009). *manLMN* has also been proposed to be involved in NAG transport in *S. pneumoniae* (Bidossi et al., 2012). Similarly, a PTS present in *nan* operon-I (putatively called *nanP*) has been suggested to play a part in the transport of glucosamine in *S. pneumoniae* (Kanehisa et al., 2014). *manLMN* and *nanP* are upregulated in our NAG-dependent transcriptome analysis, which is further confirmed by β-galactosidase assays. These observations confirm the findings of the previous studies and strengthen the involvement of *nanP* and *manLMN* in the transport of NAG.

NagA, NagB, and GlmS are very important for the metabolism of NAG and these three factors are associated with the synthesis of GlcN-6-P, a precursor for cell wall peptidoglycan synthesis in *E. coli* (Plumbridge et al., 1993; Plumbridge and Vimr, 1999). Here, we have studied the impact of *nagA, nagB*, and *glmS* deletions on the growth of *S. pneumoniae* in the presence of NAG. Our studies suggest that *nagA* and *nagB* are important for pneumococcus to grow on NAG as their deletion mutants failed to grow in the presence of NAG in the medium as a sole carbon source. NagA has also been shown to be essential for growth in the presence of NAG as a sole carbon source (Paixão et al., 2015). However, no impact of *glmS* deletion on the growth of *S. pneumoniae* was observed. In *B. subtilis, nagB* has been shown to be essential for growth in the presence of NAG (Gaugué et al., 2013). NagB and GlmS have been shown to be involved in virulence in *S. mutans* (Kawada-Matsuo et al., 2012). Inactivation of *nagB* led to a decrease in the expression of virulence factors, including cell-surface protein antigen and glucosyltransferase, and also impeded biofilm formation and saliva-induced aggregation in *S. mutans* (Kawada-Matsuo et al., 2012). Pneumococcal *nagA* mutant was tested in mouse model of colonization and of model of bronchopneumonia with bacteremia, and no difference in virulence was observed (Paixão et al., 2015). It might be still interesting to further explore the role of the *nagB* and *glmS* in virulence of *S. pneumoniae*.

In *E. coli*, a ROK-family protein (NagC) acts as a transcriptional repressor of the NAG regulon (*nagE* and *nagBACD*), which encodes genes that are involved in the uptake and metabolism of NAG. Furthermore, it has been shown that NAG binds to NagC to relieve the repression caused by NagC (Plumbridge, 1991; Titgemeyer et al., 1994). Similarly, a GntR family transcriptional regulator NagR has been shown to act as a transcriptional regulator of the genes involved in NAG utilization in *B. subtilis, S. mutans* and in some other bacteria (Bertram et al., 2011; Moye et al., 2014). In *S. mutans*, NagR has been shown to regulate the expression of *glmS* and *nagAB* by binding to the NagR operator sites called *dre* sites (Zeng and Burne, 2015). Our study suggests that NagR is present in *S. pneumoniae* and might regulate the expression of the *nagA, nagB*, and *glmS* by binding to the *dre* sites present in the promoter regions of these genes. We could not delete *nagR*, which might indicate about its essentiality or its involvement in some important cell process directly or indirectly. However, we mutated the conserved bases in the *dre* sites present in the promoter regions of *nagA, nagB*, and *glmS* which might suggest the importance of these bases in the regulation of these genes. To explore more putative *dre* sites in the D39 genome, we conducted a genome-wide search for putative pneumococcal *dre* sites. A *dre* site was only found in the promoter regions of *nagA* and *nagB*, and three *dre* sites were found in the promoter region of *glmS*. This predicted *dre* site was also found to be highly conserved in other streptococcal species as well (Novichkov et al., 2010), suggesting a similar function of NagR in these organisms.

The master transcriptional regulator, CcpA (Carbon catabolite protein A), was shown to be involved in the repression of non-preferred sugar metabolism genes in the presence of a preferred carbon source, and has a role in pneumococcal pathogenesis (Lulko et al., 2007; Zomer et al., 2007; Carvalho et al., 2011). A number of non-preferred sugar systems have also been shown to be regulated independently of CcpA by other transcriptional regulators, like the *cel* gene cluster activated by CelR in *S. pneumoniae* (Shafeeq et al., 2011). In this study, we elucidated the role of CcpA in the regulation of *nagA, nagB, glmS, manLMN*, and the *nan* operon-I by elaborating the impact of a *ccpA* deletion on the whole transcriptome of *S. pneumoniae* in the presence of NAG as a sole carbon source in CDM. Our transcriptome data demonstrated the CcpA-independent expression of *nagA, nagB*, and *glmS*, and CcpA-dependent expression of *manLMN* and the *nan* operon-I. We further analyzed the promoter regions of these genes for the presence of a *cre* box and found *cre* boxes only in the promoter regions of *manLMN* and the *nan* operon-I. The absence of *cre* boxes in the promoter regions of *nagA, nagB*, and *glmS* confirms that CcpA may not have a role in the regulation of *nagA, nagB*, and *glmS*. However, the presence of a *cre* box in the promoter regions of *manLMN* and the *nan* operon-I further supports the role of CcpA in the regulation of *manLMN* and the *nan* operon-I.

## Author contributions

Substantial contributions to the conception or design of the work; or the acquisition, analysis, or interpretation of data for the work: MA, SS, IM, BHN, and OPK. Drafting the work or revising it critically for important intellectual content: MA, SS, IM, BHN, and OPK. Final approval of the version to be published: MA, SS, IM, BHN, and OPK. Agreement to be accountable for all aspects of the work in ensuring that questions related to the accuracy or integrity of any part of the work are appropriately investigated and resolved: MA, SS, IM, BHN, and OPK.

### Conflict of interest statement

The authors declare that the research was conducted in the absence of any commercial or financial relationships that could be construed as a potential conflict of interest.
