# Supplementary material for: N-acetylglucosamine-Mediated Expression of nagA and nagB in Streptococcus pneumoniae
Source: Front Cell Infect Microbiol. 2016 Nov 16;6:158. doi: 10.3389/fcimb.2016.00158 (PMC5110562; doi:10.3389/fcimb.2016.00158)

Supplementary material:

N-acetylglucosamine-mediated expression of *nagA* and *nagB* in *Streptococcus pneumoniae*

Muhammad Afzal[1,2], Sulman Shafeeq[3], Irfan Manzoor [1,2], Birgitta Henriques-Normark[3], and Oscar P. Kuipers[1*]

1- Department of Molecular Genetics, Groningen Biomolecular Sciences and Biotechnology Institute, University of Groningen, Nijenborgh 7, 9747 AG, Groningen, The Netherlands.
2- Department of Bioinformatics and Biotechnology, Government College University, Faisalabad, Pakistan
3- Department of Microbiology, Tumor and Cell Biology, Karolinska Institutet, Nobels väg 16, Stockholm, SE-171 77, Sweden.

Table S1: Summary of transcriptome comparisons of *S. pneumoniae* D39 wild-type grown in CDM with 0.5% NAG to that grown in CDM with 0.5 % glucose and *S. pneumoniae* D39 wild-type to D39 Δ*ccpA* grown in CDM with 0.5% NAG.

[a]Gene numbers refer to D39 locus tags. [b]D39 annotation (Lanie et al., 2007), [c]Ratio represents the fold increase/decrease in the expression of genes in NAG compared to glucose. [d]Ratio represents the fold increase/decrease in the expression of genes in D39 Δ*ccpA* compared to D39 wild-type grown in CDM with 0.5% NAG.

D39 tag[a]	Function[b]	*cre* box	Ratio[c]	Ratio[d]
spd_1971	Glycosyl hydrolase-related protein	-	22.8	-
spd_0063	Beta-N-acetylhexosaminidase, StrH	ATATAATCGCTATCAA	14.2	-
spd_1970	ROK family protein	-	13.9	-
spd_1050	Tagatose 1,6-diphosphate aldolase, LacD	-	13.7	-
spd_1972	hypothetical protein	-	13.6	-
spd_0277	6- phospho-beta-glucosidase	AAGAATGCGTTTTTAT	13.1	-
spd_1969	Glycosyl hydrolase-related protein	-	11.9	-
spd_1834	Alcohol dehydrogenase, iron-containing	ATGAAACTGTTTACAA	11.8	-
spd_1051	Tagatose-6-phosphate kinase, LacC	-	11.0	-
spd_1052	Galactose-6-phosphate isomerase, LacB	-	10.7	-
spd_0090	ABC transporter, substrate-binding protein	-	10.1	-
spd_1053	Galactose-6-phosphate isomerase, LacA	-	9.9	-
spd_0089	ABC transporter, permease protein	-	7.9	-
spd_1673	Sucrose phosphorylase, GtfA	-	7.7	-
spd_0444	Endo-beta-N-acetylglucosaminidase, putative	-	7.7	-
spd_0280	Transcriptional regulator	-	7.7	-
spd_1865	Alcohol dehydrogenase, zinc-containing	ATGAAAGCCTATACTT	7.5	-
spd_0088	ABC transporter, permease protein	ATGTAATCGTTATCAA	7.5	-
spd_1677	Sugar ABC transporter, RafE	-	7.4	-
spd_0247	Glycosyl hydrolase, family protein 1	-	7.0	-
spd_0265	Alcohol dehydrogenase, zinc-containing	TGGAAAAGGCTTTCTT	6.9	-
spd_1676	Sugar ABC transporter, RafF	-	6.6	-
spd_1634	Galactokinase, GalK	GTGTAATCGTTTTCTT	6.5	-

D39 tag[a]	Function[b]	*cre* box	Ratio[c]	Ratio[d]
spd_1633	Galactose-1-phosphate uridylyltransferase, GalT	-	6.3	-
spd_0562	Beta-galactosidase precursor, BgaA	-	5.8	2.5
spd_1632	Hypothetical protein	-	5.7	-
spd_1675	Sugar ABC transporter, RafG	-	5.6	-
spd_1047	PTS system, lactose-specific IIBC components, LacE	-	5.4	-
spd_1409	Sugar ABC transporter, ATP-binding protein	TATAAAGCGTTTTCAT	5.3	-
spd_0283	PTS system, IIC component	-	5.2	-
spd_1974	Hypothetical protein	-	4.8	-
spd_1672	Hypothetical protein	-	4.8	-
spd_1678	Alpha-galactosidase AgaN	-	4.6	-
spd_0282	Hypothetical protein	-	4.4	-
spd_1046	6-phospho-beta-galactosidase, LacG	-	4.4	-
spd_1049	Transcription antiterminator, LacT	-	4.4	-
spd_1664	PTS system, trehalose-specific IIABC components	-	4.2	-
spd_0445	Phosphoglycerate kinase, Pgk	-	4.2	-
spd_0281	PTS system, IIA component	-	4.2	-
spd_1663	Alpha,alpha-phosphotrehalase, TreC	-	4.0	-
spd_1934	Maltose/maltodextrin ABC transporter, MalX	AGGAAAACGTTTGCGT	4.0	2.6
spd_0561	PTS system, IIC component, putative	-	3.9	-
spd_0279	PTS system, IIB component	-	3.9	-
spd_1495	Sugar ABC transporter, sugar-binding protein	-	3.7	4.0
spd_1973	Alpha-1,2-mannosidase, putative	-	3.6	-
spd_0068	PTS system, IID component	-	3.5	-
spd_1496	PTS system, IIBC components	-	3.4	3.7
spd_0641	Mannose-6-phosphate isomerase, ManA	-	3.4	-
spd_1588	Hypothetical protein	-	3.4	-
spd_1531	Fructokinase, ScrK	-	3.4	-
spd_0126	Pneumococcal surface protein A, PspA	-	3.2	4.1
spd_0263	PTS system, mannose-specific IIC component, ManM	-	3.2	3.4

D39 tag[a]	Function[b]	*cre* box	Ratio[c]	Ratio[d]
spd_0065	Beta-galactosidase, BgaC	ATGAAAGCGCAAACTT	3.1	-
spd_0262	PTS system, mannose/fructose/sorbose family protein, IID component, ManN	-	3.0	-
spd_0139	Glycosyl transferase, group 2 family protein	-	3.0	-
spd_0773	PTS system, fructose specific IIABC components	-	3.0	-
spd_1590	General stress protein, putative	-	3.0	-
spd_1589	Lipoprotein, putative	-	2.9	-
spd_0092	Hypothetical protein	TGTAAACGATTTCA	2.9	-
spd_0132	Hypothetical protein	-	2.8	-
spd_0140	ABC transporter, ATP-binding protein	-	2.8	-
spd_1057	PTS system, IIB component, putative	-	2.8	-
spd_1823	Glyceraldehyde-3-phosphate dehydrogenase, Gap	-	2.7	2.4
spd_1048	PTS system, lactose-specific IIA component, LacF	-	2.7	-
spd_0811	Spermidine synthase, SpeE	-	2.7	-
spd_0066	PTS system, IIB component	-	2.7	-
spd_0069	PTS system, IIA component	-	2.7	-
spd_1494	Sugar ABC transporter, permease protein	-	2.7	3.0
spd_1008	Glycogen/starch synthases, ADP-glucose type, GlgA	-	2.6	5.1
spd_1012	Phosphopyruvate hydratase, Eno	-	2.5	-
spd_1840	Hypothetical protein	-	2.5	-
spd_0067	PTS system, IIC component	-	2.5	-
spd_1635	Galactose operon repressor, GalR	-	2.5	-
spd_1493	Sugar ABC transporter, permease protein	-	2.4	3.9
spd_0138	Glycosyl transferase, group 1 family protein	-	2.4	-
spd_0772	1-phosphofructokinase, putative	-	2.4	-
spd_1302	Oxidoreductase, putative	-	2.4	-
spd_1301	NADPH-dependent FMN reductase	-	2.4	-
spd_1866	N-acetylglucosamine-6-phosphate deacetylase, NagA	-	2.4	-
spd_1846	PTS system, IIB component	-	2.3	-
spd_1007	Glucose-1-phosphate adenylyltransferase, GlgD	-	2.3	9.8

D39 tag[a]	Function[b]	*cre* box	Ratio[c]	Ratio[d]
spd_1246	glucosamine-6-phosphate isomerase, NagB	-	2.3	-
spd_0985	Phosphate acetyltransferase	-	2.2	-
spd_1300	Thiamine biosynthesis protein ApbE, putative	ATGTAAGGGCTTCAAT	2.1	-
spd_0264	PTS system, mannose-specific IIAB components, ManL	ATGAAAACGGTTTATA	2.1	3.3
spd_1504	sialidase A precursor, NanA	-	2.1	-
spd_0503	6-phospho-beta-glucosidase	-	2.1	-
spd_1175	Hypothetical protein	-	2.1	-
spd_0526	Fructose-1,6-bisphosphate aldolase	GAGAAAGGGTTTACAT	2.0	-
spd_1844	Hexulose-6-phosphate synthase, putative	-	2.0	-
spd_1797	Catabolite control protein A, CcpA	TTGAAAGTGTTTACAA	2.0	-
spd_0771	Lactose phosphotransferase system repressor, LacR	AGTGTAAACGATAACAGGA	2.0	-
spd_1492	Hypothetical protein	-	2.0	3.2
spd_1935	Maltodextrin ABC transporter, permease protein, MalC	-	2.0	2.0
spd_1932	Maltodextrin phosphorylase, MalP	-	2.0	2.0
spd_1006	Glucose-1-phosphate adenylyltransferase, GlgC	-	2.0	9.0
spd_1842	L-ribulose-5-phosphate 4-epimerase	-	2.0	-
spd_1491	Hypothetical protein	-	2.0	4.6
spd_0936	Tn5252, relaxase	-	-	25.8
spd_1747	Hypothetical protein	-	-	11.9
spd_0619	Hypothetical protein	-	-	9.9
spd_0613	Hypothetical protein	-	-	7.2
spd_0610	Hypothetical protein	-	-	6.8
spd_0634	Hypothetical protein	-	-	6.7
spd_1799	Sensor histidine kinase, putative	-	-	6.6
spd_0597	ABC transporter, ATP-binding protein	-	-	6.2
spd_0003	Hypothetical protein	-	-	6.2
spd_1800	Hypothetical protein	-	-	6.2
spd_1798	DNA-binding response regulator	-	-	5.5
spd_1748	Hypothetical protein	-	-	5.4

D39 tag[a]	Function[b]	*cre* box	Ratio[c]	Ratio[d]
spd_0595	Hypothetical protein	-	-	5.2
spd_1469	Hypothetical protein	-	-	5.0
spd_1976	Ornithine carbamoyltransferase, ArgF	-	-	5.0
spd_1005	1,4-alpha-glucan branching enzyme, GlgB	-	-	4.7
spd_1801	ABC transporter, ATP-binding protein	-	-	4.7
spd_0596	Hypothetical protein	-	-	4.7
spd_1746	Hypothetical protein	AAGAAAGGGGTTTCAT	-	4.0
spd_0612	Lipoprotein, putative	-	-	3.8
spd_1803	Hypothetical protein	-	-	3.8
spd_1752	Toxin secretion ABC transporter, ATP-binding/permease protein	TGAAATCGGTACCAC	-	3.6
spd_0611	Hypothetical protein	-	-	3.5
spd_0886	Thioredoxin family protein	-	-	3.4
spd_1753	Serine protease, subtilase family protein	-	-	3.2
spd_1979	Peptidase, M20/M25/M40 family protein	-	-	3.2
spd_1755	ABC transporter, ATP-binding protein	-	-	3.2
spd_1014	IS630-Spn1, transposase Orf1	-	-	3.1
spd_1802	Hypothetical protein	-	-	3.0
spd_1977	Carbamate kinase, ArcC	-	-	2.9
spd_0614	ABC transporter, ATP-binding protein	-	-	2.9
spd_1751	Hypothetical protein	-	-	2.8
spd_0890	Pneumococcal histidine triad protein E precursor, PhtE	-	-	2.8
spd_1754	Hypothetical protein	-	-	2.8
spd_1488	ROK family protein	ATTGAAAATGATGTCAACT	-	2.6
spd_1978	Hypothetical protein	TAGAAAGCGTTTTCTT	-	2.6
spd_0437	Hypothetical protein	-	-	2.3
spd_1360	Hypothetical protein	TATGTAACCGGTAACACAT	-	2.3
spd_1749	Bacteriocin formation protein, putative	-	-	2.3
spd_1490	Hypothetical protein	-	-	2.2
spd_0158	DNA-binding response regulator	-	-	2.2

D39 tag[a]	Function[b]	*cre* box	Ratio[c]	Ratio[d]
spd_1489	N-acetylneuraminate lyase	-	-	2.1
spd_2068	Serine protease	-	-	2.0
spd_0308	ATP-dependent Clp protease, ATP-binding subunit, ClpL	-	-	2.0
spd_1506	Acetyl xylan esterase, putative	-	-	2.0
spd_1375	NADPH-dependent FMN reductase, putative	-	-	2.0
spd_1623	Hypothetical protein	-	-	2.0
spd_0115	Hypothetical protein	-	-	-59.2
spd_0116	Hypothetical protein	-	-	-28.8
spd_0114	Hypothetical protein	-	-	-21.3
spd_0113	Hypothetical protein	-	-	-18.3
spd_0121	Hypothetical protein	-	-	-12.8
spd_0118	Hypothetical protein	-	-	-7.9
spd_0120	Hypothetical protein	-	-	-5.2
spd_0117	Hypothetical protein	-	-	-4.8
spd_0119	Hypothetical protein	-	-	-4.5
spd_1274	GMP synthase, C-terminal domain, GuaA	-	-	-3.8
spd_0112	Hypothetical protein	-	-	-3.7
spd_1742	Acetyltransferase, GNAT family protein	-	-	-3.3
spd_0105	Hypothetical protein	-	-	-3.3
spd_1438	Cadmium resistance transporter, putative	-	-	-3.2
spd_0916	Iron-compound ABC transporter, permease protein	-	-	-3.1
spd_0382	trans-2-enoyl-ACP reductase II, FabK	-	-	-3.1
spd_0122	Hypothetical protein	-	-	-3.0
spd_0380	3-oxoacyl-(acyl-carrier-protein) synthase III, FabH	-	-	-3.0
spd_0008	Septum formation initiator, putative	-	-	-2.9
spd_1394	Uncharacterized BCR	-	-	-2.9
spd_1727	Hypothetical protein	-	-	-2.8
spd_1199	Glycosyl transferase, group 2 family protein	-	-	-2.7
spd_0124	Hypothetical protein	-	-	-2.6

D39 tag[a]	Function[b]	*cre* box	Ratio[c]	Ratio[d]
spd_0383	malonyl CoA-acyl carrier protein transacylase , FabD	-	-	-2.5
spd_0378	Enoyl-CoA hydratase/isomerase family protein	-	-	-2.5
spd_0385	3-oxoacyl-[acyl-carrier-protein] synthase II , FabF	-	-	-2.4
spd_0750	Hypothetical protein	-	-	-2.4
spd_1333	Hypothetical protein	-	-	-2.3
spd_1064	Hemolysin A, putative	-	-	-2.3
spd_0407	Hypothetical protein	-	-	-2.2
spd_0751	Hypothetical protein	-	-	-2.2
spd_1364	Hypothetical protein	-	-	-2.2
spd_0145	Hypothetical protein	-	-	-2.2
spd_2051	Peptidase, M16 family protein	-	-	-2.2
spd_0123	Hypothetical protein	-	-	-2.2
spd_1726	Pneumolysin, Ply	-	-	-2.2
spd_1037	Histidine triad protein	-	-	-2.1
spd_0783	Type I restriction-modification system, S subunit, putative	-	-	-2.1
spd_0007	S4 domain protein	-	-	-2.0
spd_1907	Hypothetical protein	-	-	-2.0
spd_1100	Glucose-6-phosphate 1-dehydrogenase, Zwf	-	-2.2	-
spd_1729	Hypothetical protein	-	-2.4	-
spd_2063	Response regulator, ComE	-	-2.5	-
spd_1134	Pyrimidine operon regulatory protein/uracil phosphoribosyltransferase, PyrR	-	-2.5	-
spd_1131	Carbamoyl-phosphate synthase, CarB	-	-2.6	-
spd_1133	Aspartate carbamoyltransferase, PyrB	-	-3.0	-
spd_0448	Glutamine synthetase, GlnA	-	-3.3	-
spd_1099	Amino acid ABC transporter, ATP-binding protein	-	-3.3	-
spd_1132	Carbamoyl-phosphate synthase, CarA	-	-3.5	-
spd_0447	Transcriptional regulator, GlnR	-	-3.7	-
spd_1098	Amino acid ABC transporter, amino acid-binding protein	-	-4.4	-

Figure S1: Promoter sequences of *glmS* **(A)**, *nagA* **(B)** and *nagB* **(C)** in different Streptococci showing conservation of NagR regulatory site. Putative NagR sites are bold and underlined and translational start sites are italicized. SM= *S. mitis*, SA= *S. agalactiae*, SD= *S. dysgalactiae*, SE= *S. equi*, SG= *S. gallolyticus*, SO= *S. gordonii*, SN= *S. mutans*, SP= *S. pyogenes*, SS= *S. sanguinis*, SU= *S. suis*, ST= *S. thermophilus*, SB= *S. uberis*, PN= *S. pneumoniae*.

A

CTGTATTTTG**AAATTAGACTAGACCAATTA**AAATGTTGACAAGGATGAAATCTTGTTATATACTGATTTCACTGGTCTTTTTAGATAT**AAATTAGACTAGACCAATTT**T
TAAAGAAATGAAAGCCGATGTTAACAAACATCGTAG*GTG* --- **P*glmS* SU**

TTTTAAATGT**CAAAAAGACTATACCAATTT**TCTTGTTGACTTTATTTAAATAAAGTTATATACTGGTTCTGTTGAATTTTGGCTGTC**TATTAGACTATACCAATTA**TAA
AAAATTAAGGAGGAAGCAGTAAGGTTTTCTTACTACTAATGAAGAATT*ATG* --- **P*glmS* SN**

ACTTTTTTTA**TAATTAGACTATACCAATTT**TATGTTTGACAAGACATTGTGGATATTATATACTGTAAACATAATGTTTTTATCGGTA**TAATTAGACTATACCAATTA**T
TGA AATTATGAGAGAAAGTAGCAAGTTAACGAACTTGTTAAGGTAATTAAG*ATG* --- **P*glmS* SP**

TAGTTTTCTT**AAAGAAAACTATACCAATTT**TATACTTGACAAACCAAAAAACAGTGTGTATACTATCTTTGTTGAAGTTATTTAACTT**AAGTATACTATACCAATTT**TT
AATA AGAAGGGCTGGTTTCTAAAATATAGGAAATCAGAAGTAACGTT *ATG* --- **P*glmS* SS**

CTCATTTTTA**AAATTAGACTATACCAATAA**AATGTTTGACAAGATAAAATAGCTGGTATATACTTAAAATATAGCTTTTTGGGTAGTT**TTATTTGACTATACCAATTT**A
ATTAA TAAGAAAAGGTAGTAAGTTATTCTTACTAAGGTAAATAATA*ATG* --- **P*glmS* SB**

CAAAAAAGTT**AATTTGAACTATACCAATTT**TTGCTCTTGACAAGTGAAAG**ACAAGTATATACTGTTTT**TGCTGATTCTATAAAAGAG**GAATTAGACTATACCAATTT**TA
AGGAGAAAGCACAGCTTGCCTGTGTCGTATATACT*ATG* --- **P*glmS* SM**

CACAGAAAAT**CAAAAAGACTATACCAATTT**TTTTATTGACAAACAAAAACATTAATTATATACTGTTTTTGTTACTTTTTAACGGTAT**ATATTAATATATACCAATTC**A
AAAAGAAAGTTTAGATAGAAAGTATAAGAGAAAGCTCTTATTGGTAATAATAAT*TG* --- **P*glmS* SA**

CATAATCTGT**CGATTAGACTATACCAATTT**TGTGTTTGACAAGTAGTTGGTGATGTTATATACTGTAAACATAATATTTTTATTGG**TCTAAGGACTATACCAATTA**ATT
GAATTTTGAAAGAAAGTAGTAAGGGAACTTACTAAGGTAACTAGA*ATG* --- **P*glmS* SD**

TTGTTTCTCA**AAATATGACTATACCAATTT**TCTTGTTGACAAGATTTTTCTGAAGTTATATACTCTAAATATAACGTTTTAGTCGGTTT**ATATCTGACTATACCAATTA**
TATTGA TCATTAAAGAAAGTAGTAAGCTAGCTTACTGAGGTGTTAAGTT*TG* --- **P*glmS* SE**

TACCAATCGA**TAATTGGTATATACAAAACT**ATACCAATTTAATTGTTGACAATATAAAATCAAAGTTATATACTGATGTTGTTAGGTTTTAGAAGTCT**TTATTAGACTA**
TACCAATTTAAAAAAATA AGAAAGAATTAGCAAGTAAAGTCTTGCTAAAGGTGAAAATT*ATG* --- **P*glmS* SG**

CAATGTTGAA**TATATGGAATATACTGTCTT**CGTTAAGCTTTTCAAAGAG**CTTATTGACTATACCAATTT**TAAATGAAGGAGATATGACCTCTTTGACTTGAGGTTATTA
TTAAAAAT -*ATG* --- **P*glmS* ST**

B

CTAAAGAAGA**AATTTGGTATATACCATTTA**CAACTGTTATTAATAAGAGTATA*ATG* --- **P*nagA* SN**

TTGAATCCAA**AAAGTGGTATATACCATTTA**CAAAGTAAAGAATAAGGCGTATAATAAGGAAAAAAGGGAGGAAAAAAG*ATG* --- **P*nagA* SP**

TTTTTTGCAA**AAATAGGTATATACCATTTA**CAAATAAAAAAGAAAGAGTTATAATATAGTCAAGAAAACTCAGAGATTCAAAAGCGGGAGTTAGAAAGCAGCTATAGAAA
GATGCTAGATCTATCACAGAATTCCGTTTTGAATGGCCGAGAATATAGCATTCAAGAACAAGGGAGGCAGTCTT*ATG* --- **P*nagA* SS**

AAGCAATCCA**AAACTGGTATATACCATTTA**CAAATCTTATAATTGAGCGTATAATGGTAGAAAAAGGGAGGTTTCAA*ATG* --- **P*nagA* SB**

TCCCTCTAGA**AAATAGGTCTATACCATTTA**CAAATGAATCAGAAAGGTTTATAATGTAATTGACATAATAAATATCAAAAGTAATCTTTTAAGGAGGTCATTATT*ATG*
--- **P*nagA* SM**

ACTTGTCAAA**AAATAGGTATATACCATTTA**CAAAAATACCTTAAAGCGTTATAATGAGAGTGAATGGGGGGAAGAAG*ATG* --- **P*nagA* SA**

TTTAGGGTGG**TAAGTGGTATATACCATTTA**CAAAGTGTGGAATAAGGCGTATAATAAGGAAAAAGGGAGGAAAAAAG*ATG* --- **P*nagA* SD**

TTTAACAGAA**GGACTGGTATATACCATTTA**CAAAATAAGAATTAAGGCGTATAATAAACGAAAAAGGGAGGAAAAAAG*ATG* --- **P*nagA* SE**

AAGTTAAATA**AATTTGGTATATACCATTTA**CAAAAATAATCATTTGGCATATAATATAAGTGAATGGAGGCGGTTAAAA*ATG* --- **P*nagA* SG**

ATTTTTTGTA**AAATAGGTCTATACCATTTA**CAAATAAATTGGAAAGGTTTATAATATAGATGACATAATCAATGAGTACCAAATAGGGAGAAAGTTAGTCTATTTGGAC
AAAATC TGGAGGTTATATT*ATG* --- **P*nagA* SO**

C

TTCTTGACAA**AAATTGGTCTATACCATATA**ATAAAGAAAAAC**AAAATGGTATAGACCAAAGA**GGTGTGTT*ATG* --- **P*nagB* SU**

AGCTTGACAA**AAATTGGTCTATACCTTATACTATTTGTATAGACCGATTA**GGAGGGCGTT*ATG* --- **P*nagB* SN**

CACTTGACAA**AAATTGGTCTATACCATATA**ATAAATATAGATAGAAATGGAGGATGAAAAG*ATG* --- **P*nagB* SM**

TTATTGACAA**AAATTGGTATATACCATATA**ATATAATC**ATAAAGGTCTATACCAAATA**AAAGGAGAGTAAAC*ATG* --- **P*nagB* SA**

CTTGACAGGAAGGTTAAAAAAGAGATAATAAAG**AAAATGGTATATACCAATAT**TGAG*GTG* --- **P*nagB* ST**

Figure S2: Weight matrix of the *dre* site in the promoter regions of *glmS*, *nagA* and *nagB* from different streptococci.

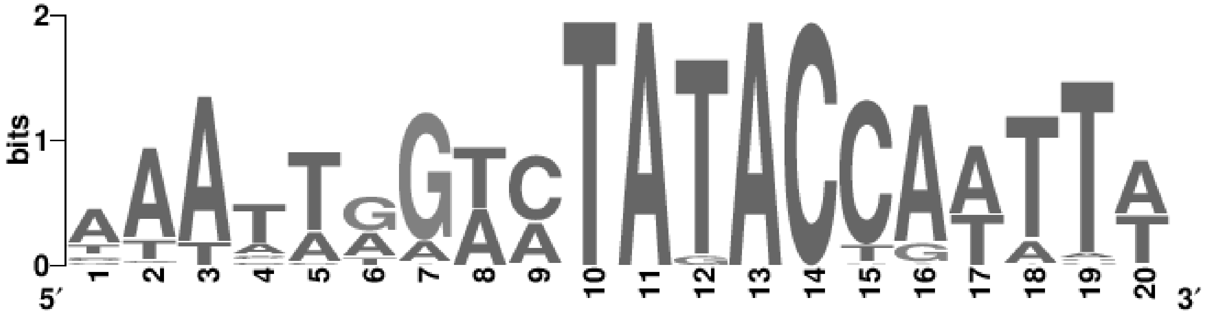

Figure S3: PCR confirmation of *nagA* (**A**), *nagB* (**B**) and *glmS* (**C**) mutants through an agarose gel.

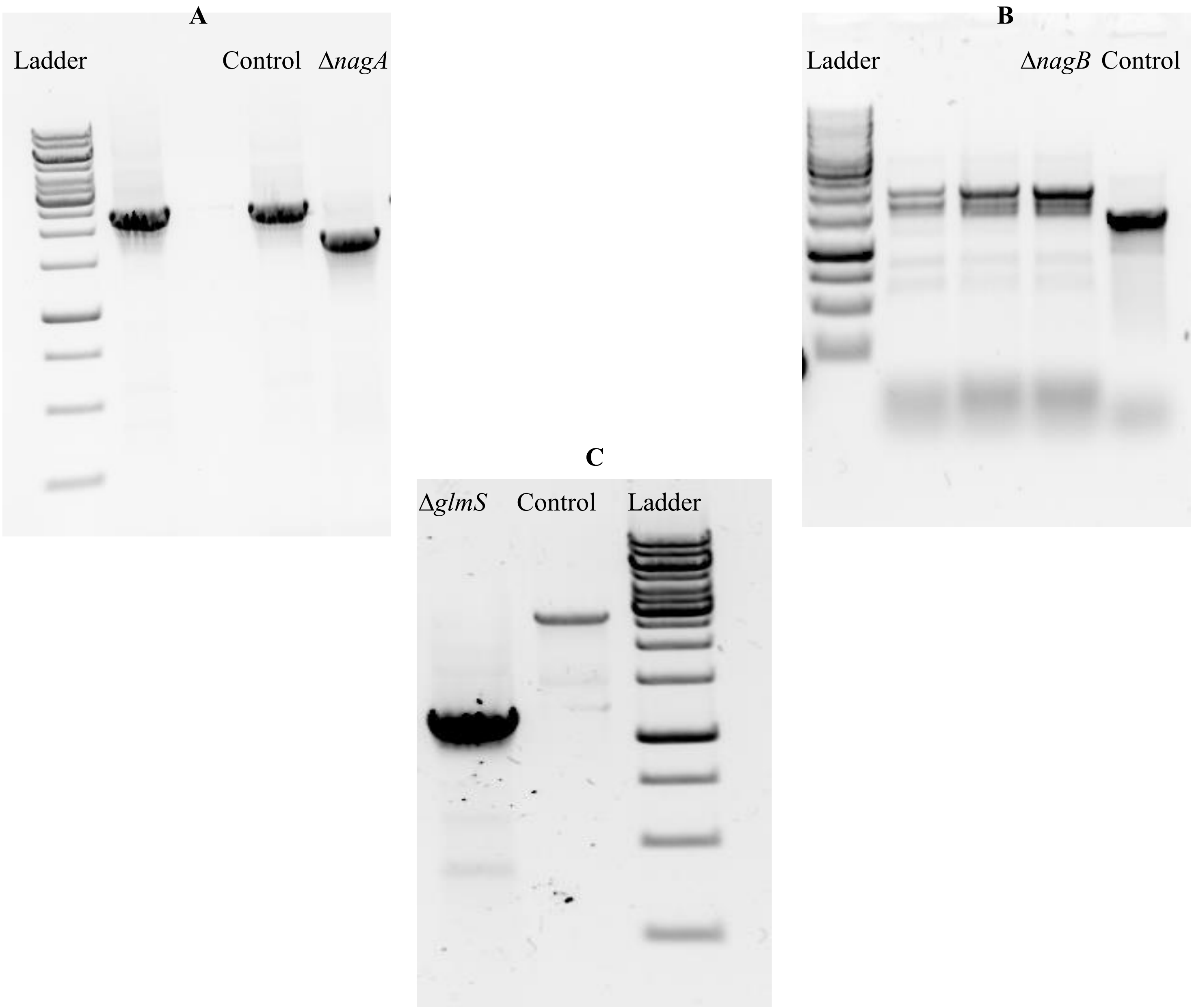

Supplement: Supplementary file 1 [file DataSheet1.pdf]
